# Arthroscopic release using F and C method versus conventional open release method in the treatment of gluteal muscle contracture: a comparative study

**DOI:** 10.1186/s12891-017-1484-6

**Published:** 2017-03-16

**Authors:** Saroj Rai, Shengyang Jin, Chunqing Meng, Nabin Chaudhary, Nira Tamang, Xiaohong Wang, Xianzhe Liu, Hong Wang, Shuhua Yang

**Affiliations:** 1 Department of Orthopedics, Wuhan Union Hospital of Tongji Medical College, Huazhong University of Science and Technology, 1277 Jie Fang Avenue, Wuhan, 430022 China; 2 Department of Radiology, Tongji Hospital of Tongji Medical College, Huazhong University of Science and Technology, 1095 Jie Fang Avenue, Wuhan, 430030 China; 3 School of Nursing, Tongji Medical College, Huazhong University of Science and Technology, 13 Hangkong Road, Wuhan, 430030 China

**Keywords:** Gluteal muscle contracture, Minimal invasive, Arthroscopic surgery, Conventional open surgery, F and C method, Intramuscular injections

## Abstract

**Background:**

Gluteal muscle contracture (GMC), a debilitating disease, usually starts in early childhood after variable dose of injections around the buttock, if left untreated it worsens gradually and persists throughout the life. Because the disease mostly affects adolescents and adults, there is always an aesthetic concerns. Purposeof the study was to introduce the arthroscopic F and C method of GMC release, and to compare its clinical efficiency with conventional open surgery in terms of clinical outcome, rate of complications, patient’s satisfactions, and recurrence.

**Methods:**

Between Jan 2013 and July 2015, 75 patients received an arthroscopic release with F and C release method and 71 patients received conventional open release of GMC. Primary surgeries in 16 years or older patients were included in the study. Two groups were compared clinically using Hip Outcome Scores – Activities of Daily Living Subscale (HOS-ADL), Hip Outcome Scores – Sports Subscale (HOS-Sports), Visual Analogue Scale (VAS), and Ye et al. evaluation criteria.

**Results:**

No statistically significant differences were observed in Hip Outcome Scores – Activities of Daily Living Subscale (HOS-ADL) (*P* = 0.078), Hip Outcome Scores – Sports Subscale (HOS-Sports) (*P* = 0.340), and Visual Analogue Scale (VAS) (*P* = 0.524) between the two groups. 74 (98.7%) patients in the arthroscopic surgery group had good to excellent results, whereas 69 (97.1%) patients in the conventional open surgery group had good to excellent results (*P* = 0.727). No statistically significant difference was observed in recurrence rate (*P* = 0.612). Statistically significant differences were observed in incision length, use of post-operative analgesia, post-operative off-bed activity, and hospital stay. Complications were significantly higher in the conventional open surgery group (*n* = 21) than in the arthroscopic surgery group (*n* = 10) (*P* = 0.016). More importantly, cosmetic satisfaction was 100% in arthroscopic release group, whereas only 71% had cosmetic satisfaction in conventional open surgery group (*P* < 0.001).

**Conclusion:**

Both, arthroscopic surgery and conventional open surgery, are highly effective tools for the GMC release in adolescent and adult patients. Arthroscopic GMC release with F and C method allows precise and selective release of contracture bands with small surgical trauma resulting fewer complications, high cosmetic satisfaction and minimal recurrence.

## Background

Gluteal muscle contracture (GMC), a debilitating disease is a clinical syndrome characterized by contracture of gluteal muscles, tensor fascia lata (TFL), iliotibial band (ITB), and related fascia, in severe cases it also involves hip external rotators and rarely the hip joint capsule [1–3]. GMC exists all across the globe but is more prevalent in China, with childhood incidence rate of 1–2.5% [4–8]. It is associated with intramuscular injections of antibiotics and antimalarial agents like quinine into buttocks [9–12].

**Fig. 1:**
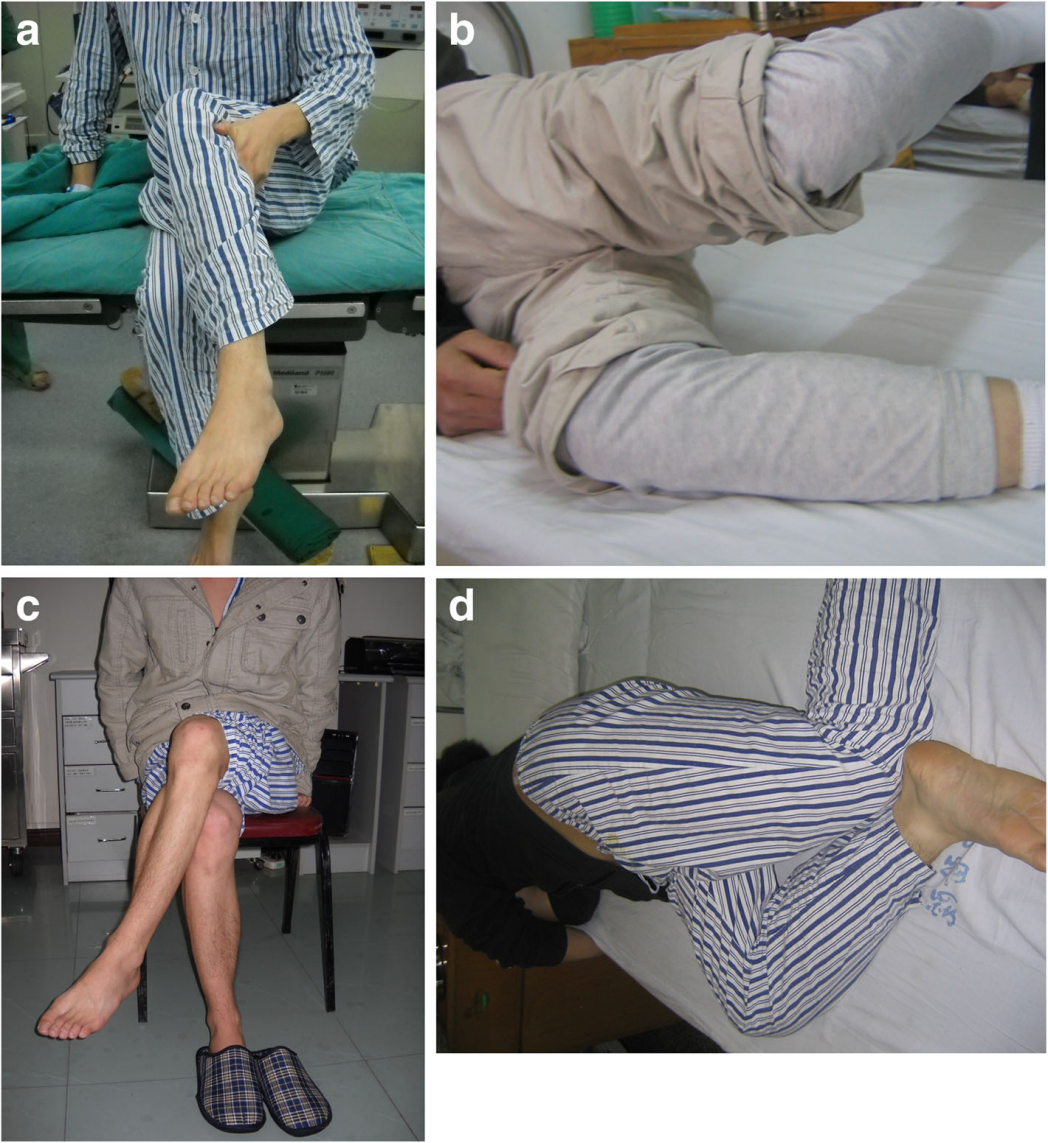
Arthroscopic release of bilateral gluteal muscles contractures. **a** Pre-operatively, patient was unable to cross the legs completely, and **b** Ober’s sign was positive. **c** 3 days post-operative pictures, patient was able to cross the leg completely without any support, and **d** Ober’s sign was negative

Pathognomonic presentation of the disease is abduction and external rotation along with limited flexion and adduction of affected hip [1]. Other features include difficulty in crossing or overlapping the legs (cross sign) (Fig. 1a) and squatting (squatting test), positive Ober’s sign (Fig. 1b), frog leg sign, out-toeing gait, flattened and cone shaped buttock, apparent leg length discrepancy, pelvic obliquity, snapping sound, and a compensatory lumbar scoliosis [13].

GMC usually starts in early childhood, if left untreated it worsens gradually and persists throughout the life [14]. Because the disease mostly affects adolescents and adults, there is always an aesthetic concern. For long, the conventional open release was regarded as the gold standard treatment method for GMC; however, the high rate of complications such as hypertrophic scar, post-operative adhesion and sciatic nerve injury tremendously decreased the patient’s satisfactions [15, 16]. Recently, arthroscopic release of GMC has been introduced as a minimally invasive technique and has dramatically gained popularity among orthopedic surgeons. It has been reported that it avoids extensive surgical trauma resulting in minimal complications in comparison to the conventional open surgery. It has high level of patient’s satisfactions and has excellent clinical outcome [4]. However, previous literatures regarding the comparison of surgical outcomes and complications of these two surgical procedures are still scarce. Currently, no standardized arthroscopic surgical technique for GMC release exists which can address the pathology in a systematic way.

In this study, we performed arthroscopic release and conventional open release of GMC in adolescents and adult populations. The main purpose of this study was to introduce the arthroscopic F and C method of GMC release, and to compare its clinical efficiency with conventional open surgery in terms of subjective and objective clinical outcomes, patient’s satisfactions, complications and recurrence. Our hypothesis was that the arthroscopic release of GMC using F and C method would provide exceptionally precise and selective release of contractures which would improve the clinical outcomes, thus decreasing the complications associated with conventional open surgery.

## Methods

### Patients

Between Jan 2013 and July 2015, 167 consecutive patients with GMC underwent surgical release using either arthroscopic technique or conventional open release technique. All the patients were carefully examined in the clinic and pre-operatively under anesthesia on the operating table by the senior surgeon in order to determine the severity of disease.

Inclusion criteria involved primary GMC releases of 16 years or older patients who could complete the study and the strict rehabilitation protocol. Out of 167 patients, 146 patients provided written informed consent and were included in the study. Every patient was clearly informed about the disease conditions and the surgical procedures along with its benefits and risks. The types of procedures were selected according to the surgeon’s recommendations and patient’s choice, and were performed by or under direct supervision of senior surgeons HW and SHY. 75 patients (150 hips) (male = 25, and female = 50) with the mean age of 25.05 years (16 to 46 years) received the arthroscopic release and 71 patients (142 hips) (male = 33, and female = 38) with the mean age of 25.30 years (17 to 42 years) received the conventional open release. All the patients were classified according to Zhao et al. classification system [16]. In the arthroscopic surgery group, 25 patients were classified as mild, 40 as moderate, and 10 as severe diseases, whereas in the conventional open surgery group, 13 patients were classified as mild, 41 as moderate, and 17 as severe disease.

## Surgical procedure

### Conventional open surgery

**Fig. 2:**
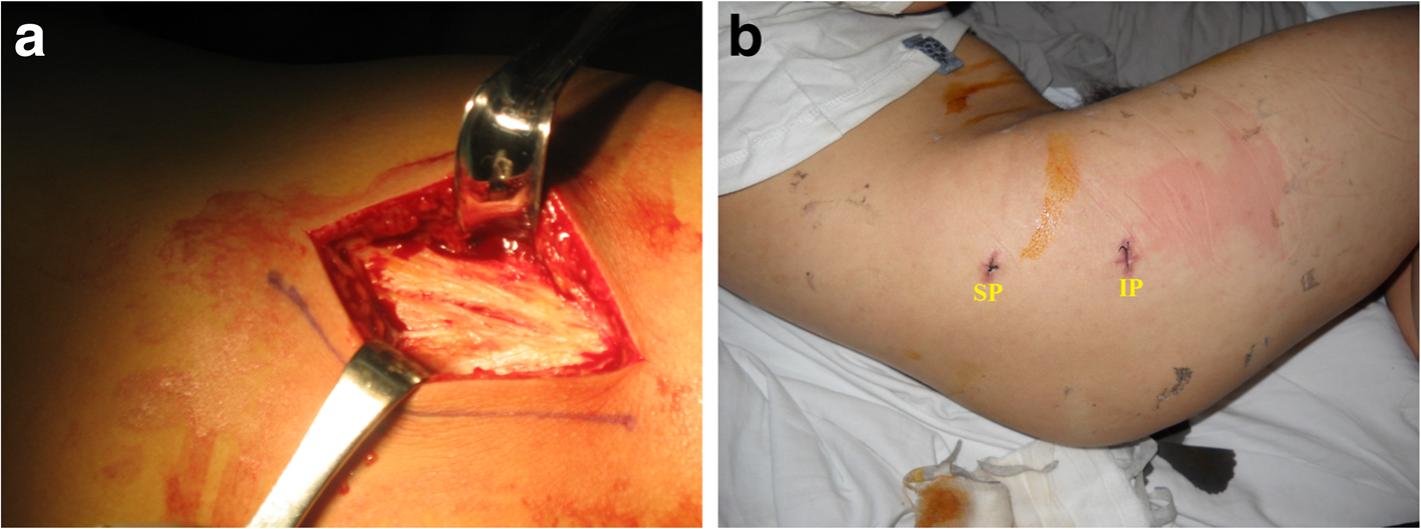
Comparison of incision sizes of GMC release. **a** shows a big longitudinal incision, which was made for the conventional open surgery, and **b** shows 2 tiny incisions made for the arthroscopic 2 portal technique

Variable lengths and shapes of skin incisions (5 cm −10 cm) were made in the lateral position over buttock and greater trochanter, followed by division of contracture bands (Fig. 2a). Contractile fibrotic bands were divided in a sequential order according to the muscle group involvement, (iliotibial bands, gluteus maximus, gluteus medius, gluteus minimus, piriformis and even hip joint capsule) starting from superficial to deeper structures until all the signs and symptoms completely disappeared intra-operatively. Any residual deformities were meticulously assessed, and complete release of contracture was confirmed by adduction, flexion, internal rotation, Ober’s sign, cross leg sign, and palpable click. Finally, appropriate haemostases were maintained, wounds were irrigated with normal saline, a drainage tube placed, and wounds were closed.

### Arthroscopic surgery (F and C method)

The procedure involved marking of all the anatomical landmarks, like greater trochanter (GT), anterior and posterior boarders of contracted glutei, and most importantly the course of the sciatic nerve in neutral lateral position of the hip. Two portals were usually made and sometimes three portals according to variations in the location and depths of GMC groups. First viewing portal (0.5 cm) was made just over the centre of GT (Fig. 2b). An artificial working space (6 cm × 8 cm) was created in the interval between the subcutaneous fascia and the contracture bands using curette. Silvery white contracture bands were visible when an arthroscope was introduced into the artificial space filled by continuous irrigation of normal saline. About 10 cm above the first portal in the longitudinal axis, second working portal was made under arthroscopy. Any fatty and fibrous tissues in the artificial space were meticulously removed by a shaver and a radio-frequency ablation device. There was always a chance of bleeding from muscles, which was prevented by the prophylactic use of adrenalin (1 mg in 3 l) in a continuous flow of saline, and any visible bleeders were coagulated instantly.

**Fig. 3:**
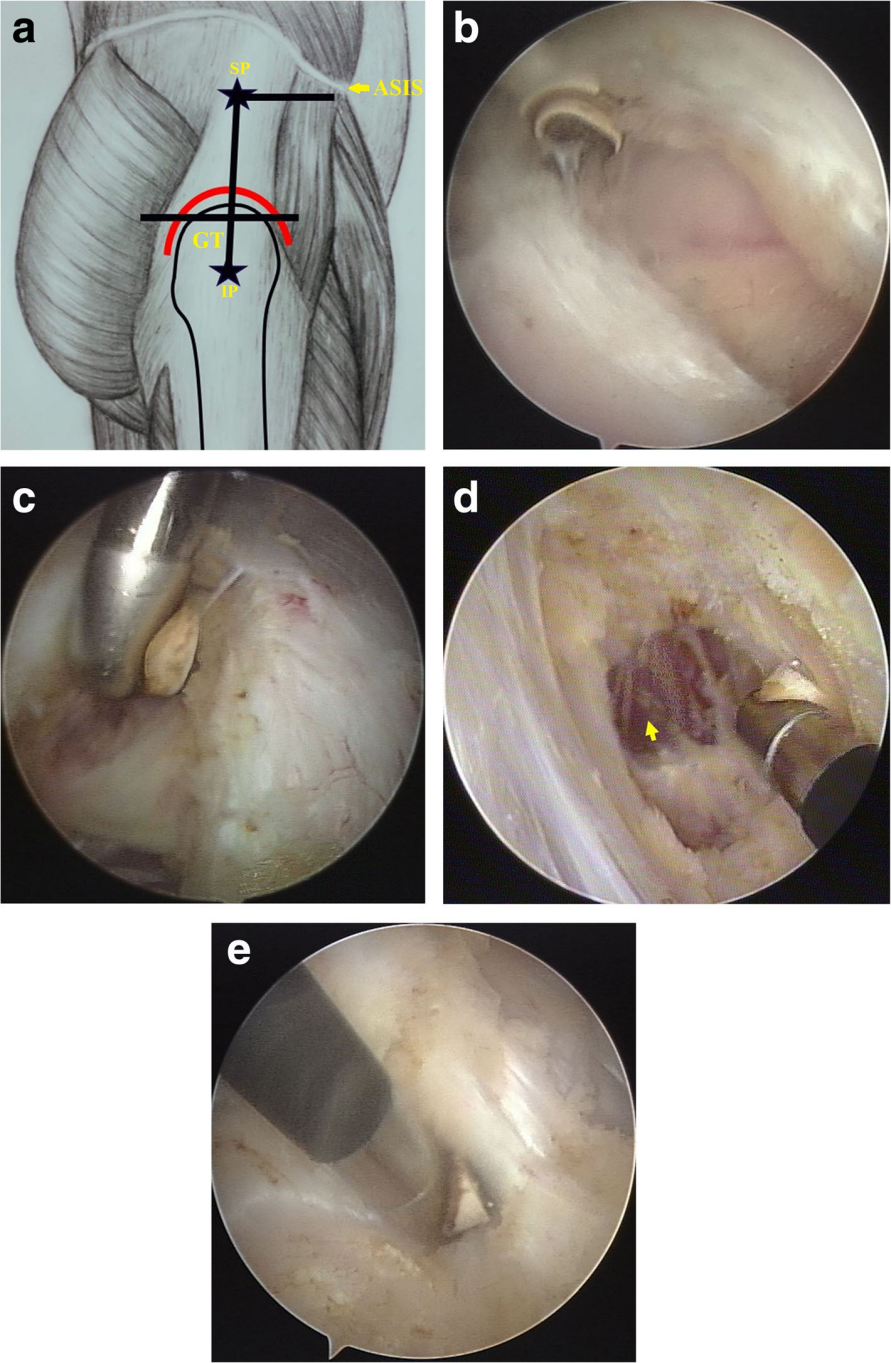
Arthroscopic release of GMC using F and C method. **a** Represents the schematic outline of F (*Black*) and C (*Red*) method, where SP, IP, GT and ASIS represent superior portal, inferior portal, greater trochanter and anterior superior iliac spine, respectively. **b**, **c**, and **d** are intra-operative pictures showing the F release method, division is started from the centre of GT and continued up to the ASIS to divide iliotibial band, tensor fascia lata and gluteus maximus muscles. A *yellow arrowhead* in the picture **d** represents a healthy gluteal muscle. **e** is an intra-operative picture of the C release method to divide gluteus medius, gluteus minimus and deeper structures around GT

Division of contracture bands using a radio-frequency ablation device was then performed using F and C method (Fig. 3a). Initially, division of the ITB was started from the centre of GT (approx. 4 cm below the superior pole of GT) and continued superiorly up to about 10 cm in the longitudinal axis (Fig. 3b). Then, the radio-frequency ablation device was faced anteriorly to divide contractures of tensor fascia latae (TFL), and continued up to the anterior superior iliac spine (ASIS) (Fig. 3c). Gluteus maximus contractures were then divided transversely from approximately 1 cm below the superior pole of GT until silvery white bands of contractures were visible, which completed the F shaped release of GMC (Fig. 3d). The arthroscopic instruments were then advanced further deep to visualize the contractures of gluteus medius, gluteus minimus and deeper structures, and were divided around the GT in the C shaped fashion (Fig. 3e). Finally, complete division of contracture bands were meticulously assessed using same technique as in the conventional open surgery.

### Rehabilitation protocol

Drainage tube was placed routinely, and removed 24 to 48 h after surgery. Rehabilitation protocols were similar for both the groups, patients were instructed to do the functional exercises after elimination of post-surgical pain, or after the drainage tube was removed. At first, patients were placed in continuous passive motion (CPM) machine to allow passive hip and knee flexion exercises, followed by an active range of motion (ROM) exercises, then allowed to walk, and gradually to perform other exercises including crossing legs, straight walking, crouching with closed knees (Fig. 1c & d). Sutures were removed in 2 weeks for both the groups.

### Patient’s evaluation

All the patients were followed up for at least 18 months (mean, 22 months). Patients were clinically assessed by subjective and objective evaluations. Subjective evaluations were performed using hip outcome scores (HOS), which assesses activities of daily living (HOS-ADL) and sports activities (HOS-Sports), and a visual analogue scale (VAS) for pain [17]. Objective clinical evaluation was performed using evaluation criteria set by Ye et al. (2012) [14]. It includes 4 parameters. First, closing knees together while squatting and standing: 3 points were given if the patient could squat and stand freely, 2 points if the patient could squat and stand partly with help, 1 point if the patient could squat and stand wholly with help and 0 point if the patient was unable to stand or squat. Second, crossing and overlapping the legs with 90° of hip and knee flexion: 3 points were given if the patient could cross and overlap the legs freely, 2 points if the patient could cross or overlap the legs partly with help, 1 point if the patient could cross or overlap the legs wholly with help, and 0 point if the patient was unable to cross and overlap the legs. Third, ambulation: 2 points were given if the patient did not have trendelenburg gait involuntary, 1 point if the patient had no trendelenburg gait consciously, and 0 point if the patient had trendelenburg gait consciously. Fourth, glide of fibrotic bands in the iliotibial tract: 2 points were given if the patient had no gliding of fibrotic band and no resistance, 1 point if the patient had gliding of fibrotic band and resistance could be felt, and 0 point if the patient had no fibrotic band, but resistance could be felt. Clinical grade was considered to be excellent if the points obtained was 9–10; good if 7–8; and poor if 0–6. A self administered questionnaire for patients’ satisfaction in terms of cosmetic and functional satisfaction was carried out and graded as satisfied or dissatisfied. Other parameters included incision lengths, duration of surgery, post-operative analgesia, off-bed activity time, complications, and recurrence.

### Statistical analysis

We used Statistical Package of Social Sciences (IBM SPSS Statistics 23) version 23 for statistical analysis. Categorical data were analyzed using Chi-square test and Fisher’s test, and independent *t* test (two tailed) was chosen for analysis of parametric continuous data, whereas the Mann-Whitney *U* test was used to compare non parametric continuous data. Results of categorical data were presented as frequencies and percentages, whereas results of continuous data were presented as mean ± standard deviation (SD). Statistical differences were considered significant for *P* values <0.05. A post hoc power analysis was performed using HOS and Ye et al. evaluation criteria as primary outcome measures.

## Results

### General results

Demographic characteristics of patients are well illustrated in Table 1. The average duration of procedure in one side was 21.75 min (8–55) in the arthroscopic surgery group and 19.32 min (9–55) in the conventional open surgery group (*P* = 0.066). The average length of incision was 0.52 cm (0.5 - 1) for the arthroscopic surgery group, and was 7.18 cm (5–10) for the conventional open surgery group (*P* < 0.001). Post-operative analgesia was not needed for 67 (89.3%) patients, and 8 (10.7%) patients needed in the arthroscopic surgery group, whereas in 21 (29.6%) patients it was not needed, and 48 (67.6%) patients it was needed in the conventional open surgery group (*P* < 0.001). The average post-operative hospital stay for the arthroscopic surgery group was 3.56 days, and that for the conventional open surgery group was 5.23 days (*P* < 0.001). Similarly, average post-operative off-bed activity was 1.6 days for the arthroscopic surgery group, and 3.75 days for the conventional open surgery group (*P* < 0.001). There were also no statistically significant differences between ages, gender and severity of disease.

**Table 1:** Comparison of patient’s demographic characteristics of two surgical options (mean ± SD or *n*, %)

Parameters	Arthroscopic surgery (*n* = 75)	Conventional open surgery (*n* = 71)	*P*-value
Age	25.07 ± 6.19 (16–46)	25.30 ± 5.38 (17–42)	0.812
Male/Female (*n*)	25/50	33/38	0.105
Zhao Classification			0.064
Mild	25 (33.4)	13 (18.3)	
Moderate	40 (53.3)	41 (57.7)	
Severe	10 (13.3)	17 (23.9)	
Duration of procedure (min)	21.75 ± 8.23 (8–55)	19.32 ± 7.54 (9–45)	0.066
Incision length (cm)	0.52 ± 0.09 (0.5 - 1)	7.18 ± 1.24 (5–10)	<0.001*
Follow-up (months)	29.40 ± 6.82 (18–42)	26.76 ± 6.03 (18–40)	0.015*
Post operative analgesia (*n*, %)			<0.001*
Not required	67 (89.3)	21 (29.6)	
Required	8 (10.7)	48 (67.6)	
Post-operative hospital stay (days)	3.56 ± 0.70 (3–5)	5.23 ± 1.00 (4–7)	<0.001*
Post-operative off-bed activity (days)	1.60 ± 0.65 (1–3)	3.75 ± 0.84 (2–6)	<0.001*

*SD* Standard Deviation, *min* minute, *cm* centimeter; (*) = Statistically significant difference between the groups

### Clinical results

**Fig. 4:**
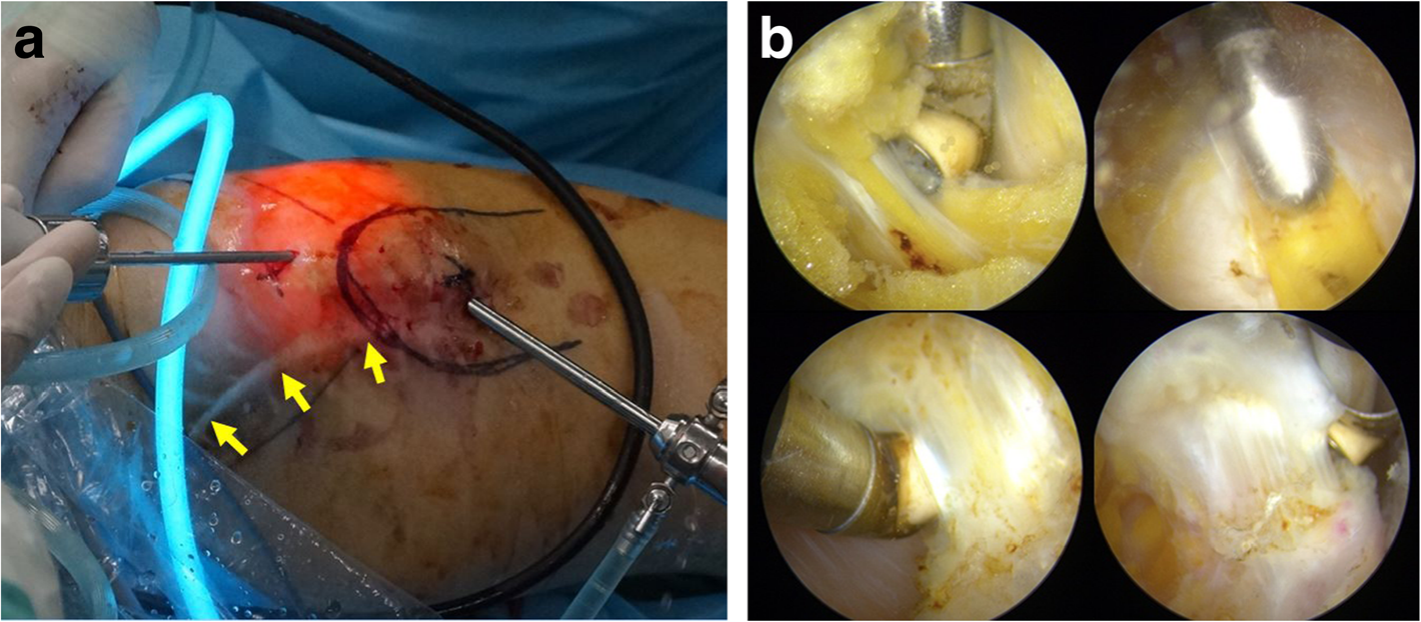
Revision GMC release. **a** Arthroscopic release of a recurrent GMC which was previously operated with conventional open surgery, where a big surgical scar can be seen (*arrows*). **b** Arthroscopic pictures showing massive adhesion of contractures

No statistically significant difference between the two groups were observed according to HOS-ADL (*P* = 0.078) and HOS- Sports (*P* = 0.340) Subscales (Table 2). 71 (94.7%) patients in the arthroscopic surgery group and 64 (90.1%) patients in the conventional open surgery group rated 90% or more functions during the usual activity of daily living, remaining patients in each group rated 80% or more (*P* = 0.300). Similarly, 70 (93.3%) patients in the arthroscopic surgery group and 63 (88.7%) patients in the conventional open surgery group rated 90% or more functions during the usual sports activities, remaining patients in each group rated 80% or more following surgery (*P* = 0.329). Ye et al. evaluation showed excellent result in 71 (94.7%), good in 3 (4.0%) and poor in 1 (1.3%) patients in the arthroscopic surgery group, whereas excellent in 65 (91.5%), good in 4 (5.6%) and poor in 2 (2.8%) patients in the open surgery group (*P* = 0.727). Moreover, 100% patients in the arthroscopic surgery group, and only 51 patients in the conventional open surgery group had cosmetic satisfaction (*P* < 0.001). Similarly, 94.7% patients in the arthroscopic group, and 93% patients in the conventional open surgery group had functional satisfaction (*P* = 0.740). No significant difference was observed in the recurrence rate (*P* = 0.612). One patient in the arthroscopic surgery group with severe disease was considered to be recurrent, but she refused second operation as she could perform all the activities of daily living normally. While 2 patients in the conventional open surgery group had recurrence, and underwent an arthroscopic release. Arthroscopic release of recurrent GMC revealed severe adhesion of scar tissues (Fig. 4); however, both the patients reported excellent outcome. In VAS scale, no any pain was reported by 74 (98.7%) patients in the arthroscopic surgery group, and 69 (97.2%) patients in the conventional open surgery group (*P* = 0.524) (Table 3). Post hoc power analysis revealed power of 43, 16, and 14% as HOS-ADL subscale, HOS-Sports subscale and Ye et al. evaluation criteria, respectively. This shows that a very large number of patients would have been needed to detect difference between two groups.

**Table 2:** Clinical outcomes of patients with gluteal muscle contracture release with two surgical options (mean ± SD or *n*, %)

Parameters	Arthroscopic surgery (*n* = 75)	Conventional open surgery (*n* = 71)	*P*-value
HOS - ADL Subscale	97.98 ± 3.98 (84–100)	96.64 ± 5.07 (81–100)	0.078
HOS - Sports Subscale	95.55 ± 4.83 (81–100)	94.67 ± 6.17 (75–100)	0.340
Ye et al. evaluation criteria (*n*, %)			0.727
Excellent	71 (94.7)	65 (91.6)	
Good	3 (4)	4 (5.6)	
Poor	1 (1.3)	2 (2.8)	
Patient satisfaction (*n*, %)			
Cosmetic satisfaction	75 (100)	51 (71.8)	<0.001*
Functional Satisfaction	71 (94.7)	66 (93)	0.740
Recurrence (*n*, %)			0.612
No	74 (98.7)	69 (97.2)	
Yes	1 (1.3)	2 (2.8)	

No statistical significant differences were observed in all the clinical parameters except cosmetic satisfaction

*SD* Standard deviation, *HOS-ADL* Hip Outcome Score - Activity of daily living, *HOS-Sports* Hip Outcome Score – Sports

*Statistically significant difference between the groups

**Table 3:** Visual analogue scale (VAS) score of two surgical options (*n*, %)

VAS score	Arthroscopic surgery (*n* = 75)	Conventional open surgery (*n* = 71)
0	74 (98.7)	69 (97.2)
1	1 (1.3)	2 (2.8)

*VAS* Visual Analogue Scale

### Complication

Complications of the conventional open surgery group was significantly higher than the arthroscopic surgery group (*P* = 0.016) (Table 4). In the arthroscopic surgery group, 2 post-operative minimal hematomas and 5 bruising were observed, but no intervention was needed. 3 patients had a positive trendelenburg gait, but were relieved within 6 months period. No wound infection, no sciatic nerve injury, and no hypertrophic scar were observed in this group. In the conventional open surgery group, 3 post-operative hematomas and 6 bruising were observed as early complications, 2 painful hematomas needed surgical evacuation. 2 patients had a positive trendelenburg gait, but were relieved within the 6 months period. A patient had transient sciatic nerve palsy. 5 patients had hypertrophic scars, and 4 patients had adhesions around the buttocks. No other complications occurred in conventional open surgery group.

**Table 4:** Comparison of rate of complications between two surgical options

Complications	Arthroscopic surgery (*n* = 75)	Conventional open surgery (*n* = 71)
Hematoma	2	3
Bruising	5	6
Superficial infection	0	0
Transient sciatic nerve palsy	0	1
Permanent sciatic nerve palsy	0	0
Trendelenburg gait	3	2
Hypertrophic scar	0	5
Band/Adhesion	0	4
Total	10	21

## Discussion

The most important finding of our study was that the arthroscopic release of GMC is as good as the conventional open surgical release with significantly minimal complications in adolescents and adults. At the final follow up, both the groups had excellent subjective as well as objective clinical outcomes with good to excellent results in 74/75 (98.7%) in the arthroscopic surgery group and 69/71 (97.1%) in the conventional open surgery group. Most importantly, the cosmetic satisfaction was 100% in the arthroscopic surgery group.

The treatment options of GMC include non-operative management and operative management, followed by a programmed rehabilitation. The non-operative management includes massage, physiotherapy, shortwave diathermy, and active and passive stretching exercises [16], and is recommended only for the mild cases or for those patients who are ineligible to undergo surgical release; however the end result is disappointing. Zhao et al. (2009) demonstrated that the non-operative management was successful only in 38% among 49 patients regardless of a very strict rehabilitation protocol [16].

The surgical release is the gold standard treatment option for an established GMC, and is recommended for all the patients who are motivated to comply with a strict post-operative rehabilitation program [16, 18]. Various surgical options are available for GMC release including the conventional open surgery and the minimal invasive arthroscopic surgery. However, the choice of surgery is truly reliant on the severity of the disease, availability of experts and highly sophisticated tools. Early post-operative rehabilitation plays a key role for the rapid recovery, reduction of complications, and attainment of optimum outcome [13]. Despite having a good hip ROM intra-operatively, outcome was poor in patients who had a poor compliance [18]. In our study, we encouraged all the patients to have passive and active ROM exercises from the very next day following operation, followed by a programmed rehabilitation.

The conventional open surgery is being performed since decades, and is indicated in all the established GMCs, but is highly recommended for the severe cases because wide skin incision provides an adequate exposure allowing the division of fibrotic bands under the direct vision [13]. Multiple previous studies reported excellent outcomes of open release; however, a large surgical trauma significantly increases the risk of post-operative complications like acute painful hematoma, bruising, wound infection, hypertrophic scar formation, wound dehiscence, unsteadiness in walking and neurovascular injury [15, 16]. Zhao et al. (2009) reported 83% excellent result with the open surgery in their case series of 129 patients, although they reported 64 cases of hypertrophic scar in moderate and severe disease, some exceeding 7 mm, 4 hematomas, 2 infections and one wound dehiscence [16]. Moreover, in a retrospective case series of 428 patients, 98.5% patients had good to excellent outcome but 16 patients reported unsteadiness in walking, and 6 patients under 5 years had a fair result due to the poor compliance [18]. Reports suggested that the patients who underwent Z-lengthening of contracture bands, especially ITB required extended rehabilitation to achieve full range of active hip motion [19]. Despite having excellent outcome with the conventional open surgery, these well-known complications cause a negative effect on patients’ clinical outcomes as well as cosmetic satisfactions, particularly in youth.

In 2009, Liu et al. introduced an arthroscopic release of GMC as a new and minimally invasive surgical release technique as arthroscopy guided release could avoid extensive surgical trauma by a precise and selective contractures release in an extremely controlled manner [4]. Their hypothesis was that the arthroscopic release of GMC using radio-frequency energy would decrease the complications associated with open surgery and would provide adequate hip adduction and flexion ROM [4]. They reported a tremendous improvement in the hip joint adduction from 10.4° to 45.3°, flexion from 44.8° to 110.2° and correction of out toe gaits with different degrees without associated complications related to open surgery in an average follow-up of 17.4 months in 150 patients [4].

Our findings were consistent with the findings of Fu et al. (2011) [20], who compared the endoscopic surgery with the traditional open surgery in children. They demonstrated a significant superior result with endoscopic release in terms of small surgical trauma, less post-surgical pain, early off-bed activity, short hospital stay and cosmetic satisfaction, but there were no statistical differences in duration of surgery, complication, clinical outcome, and recurrence rate [20]. However, they reported conversion of endoscopic release to open procedure in 4 cases of Level 3 disease with a large contracture [20]. The conversion of endoscopic surgery was possibly because of failure to address the contractures with a systematic approach leading to incomplete division of large GMC, or involvement of deeper structures which was difficult to visualize under arthroscopy. In our study, we did not convert any cases from arthroscopic surgery group to conventional open surgery. Complete division was possible due to meticulous pre-operative identification of the involved tissues by thorough physical and radiological examinations, and application of the technique that we developed as F and C method of contracture release under arthroscopy. To our knowledge, the F and C method is the first standardized arthroscopic surgical release technique of GMC. We believe, this method allows surgeons to have an accurate identification of pathology intra-operatively, and precise and very selective division of contracture bands in a systematic way in all the directions.

The hypertrophic scars and band formations were extremely notorious and inevitable delayed complications of open surgery, leading to aesthetic dissatisfactions in young patients [15, 16]. In our study, 9 patients in the conventional open surgery group had hypertrophic scars. Meticulous intra-operative aseptic precautions resulted in no infection in all cases of GMC. On the other hand, post-operative hematoma and bruising were the leading immediate complications, 2 large acute painful hematomas in the conventional open surgery group needed surgical evacuation. Hematoma occurred in relatively inactive patients. We considered the lack of early post-operative rehabilitation to be the cause of hematoma. More importantly, post-operative functional exercise permits reduction of pain and swelling, and the hip muscles to strengthen. Therefore, it should be started as early as possible in order to maintain an intra-operative ROM which is essential to prevent the development of hematoma, to attain optimal outcome, and to minimize the recurrence. Thus, delaying rehabilitation might lead to severe morbidity and cosmetic dissatisfaction to the patients.

Strength of our study is in the fact that it is the first ever comparative study of arthroscopic release using F and C method and conventional open surgical release in adolescent and adult populations. It showed comparable or even better result than in pediatric population by Fu et al. (2011) [20]. However, our study has several known limitations. First, all biases related with a retrospective, non-randomized study persuade the interpretation of our results. Our study is particularly subject to selection bias as the decision on surgical option was at the discretion of the chief operating surgeons. The results presented in this study are from a single hospital, and may also reflect regional and institutional bias. Second, a post hoc power analysis showed under power clinical results (for acceptable power of 80% in 0.05 significant level). This is likely due to inadequate sample size, in our case as GMC is a relatively rare pathology. Consecutive cases of GMC were taken within the given duration resulting in small sample size, thus affecting the statistical power.

## Conclusion

Both the arthroscopic surgery and conventional open surgery are highly effective tools for GMC release in adolescent and adult patients. But, further study with an adequate sample size is required to conclude whether statistical significant difference exists between two surgical options or not. However, arthroscopic release of GMC using F and C method allows precise and selective release of contracture bands with small surgical trauma resulting in fewer complications, high cosmetic satisfactions and negligible recurrence. A careful assessment of the severity of disease, patient selection, and thorough knowledge about instrumentation and advanced surgical skills are extremely essential. For a big and complex GMC, conventional open release should always be reserved, and surgeons must not try to avoid the option just because of interest in performing an arthroscopic release.

## Abbreviations

CPM: Continuous passive motion
GMC: Gluteal muscle contracture
HOS: Hip outcome score
ITB: Iliotibial band
ROM: Range of motion
SD: Standard deviation
TFL: Tensor fascia lata
VAS: Visual analogue scale
