# Correction to: Arthroscopic release using F and C method versus conventional open release method in the treatment of gluteal muscle contracture: a comparative study

**DOI:** 10.1186/s12891-018-2119-2

**Published:** 2018-06-07

**Authors:** Saroj Rai, Shengyang Jin, Chunqing Meng, Nabin Chaudhary, Nira Tamang, Xiaohong Wang, Xianzhe Liu, Hong Wang, Shuhua Yang

**Affiliations:** 1 Department of Orthopaedics, Union Hospital, Tongji Medical College, Huazhong University of Science and Technology, Wuhan, 430022 China; 2 Department of Radiology, Tongji Hospital of Tongji Medical College, Huazhong University of Science and Technology, 1095 Jie Fang Avenue, Wuhan, 430030 China; 3 School of Nursing, Tongji Medical College, Huazhong University of Science and Technology, 13 Hangkong Road, Wuhan, 430030 China

## Correction

Upon publication of this article [1], it was requested that: the corresponding author, Hong Wang’s affiliation address be changed from:

‘Department of Orthopedics, Wuhan Union Hospital of Tongji Medical College, Huazhong University of Science and Technology, 1277 Jie Fang Avenue, Wuhan 430022, China’.

To:

Department of Orthopaedics, Union Hospital, Tongji Medical College, Huazhong University of Science and Technology, Wuhan 430,022, China.

The affiliation address has been changed by means of this Correction article.
